# Equitable Digital Frailty Screening for Marginalized Older Adults Using Audio Computer-Assisted Self-Interview: Collaborative Development Guide and User Testing Study

**DOI:** 10.2196/85768

**Published:** 2026-05-13

**Authors:** Jane Ye In Hwang, Tony Butler, Peter Schofield, Rhys Mantell, Michael Kasumovic, Natasha Ginnivan, Kylie Radford, Ruth E Hubbard, Emily Gordon, Arthur Montalto, Phillip Snoyman, Adrienne Withall

**Affiliations:** 1 School of Population Health, UNSW Sydney, Building C29 HTH Level 05, 55 Botany Street Randwick, UNSW, 2052, Australia, 61 93480073; 2 University of Newcastle Australia, Newcastle, Australia; 3 School of Biological, Earth & Environmental Sciences, Faculty of Science, UNSW Sydney, UNSW, Australia; 4 Neuroscience Research Australia, Sydney, Australia; 5 School of Psychology, UNSW Sydney, UNSW, Australia; 6 Australian Frailty Network, The University of Queensland, Woolloongabba, Australia; 7 Department of General and Geriatric Medicine, Princess Alexandra Hospital, The University of Queensland, Woolloongabba, Australia; 8 NSW Department of Communities and Justice, Corrective Services NSW, Sydney, Australia

**Keywords:** frailty, digital health, health screening, health equity, co-design, multidisciplinary

## Abstract

**Background:**

Older adults facing social or structural marginalization for reasons such as lower literacy, digital exclusion, financial constraints, restricted living environments, or complex health histories, face persistent barriers to much-needed health screening. Digital health tools, particularly those using audio computer-assisted self-interview (ACASI) technology, offer potential to overcome these barriers (audio-delivered and self-administrable), but their application to marginalized populations remains underexplored. Moreover, guidance is limited for developing such tools which require collaboration within cross-disciplinary teams. This paper presents development insights and user testing findings from the ASCAPE (Audio App-Delivered Screening for Cognition and Age-Related Health in Prisoners) project, which aimed to develop equitable digital frailty and cognition screening for older people in Australian prisons.

**Objective:**

This study aims to describe the collaborative development of the “ASCAPE-HS” prototype, a tablet-based ACASI-delivered Frailty Index and aging screener, and to synthesize key lessons from the project that can inform equitable digital health tool development in hard-to-reach older adults. Also, to present findings on the usability and acceptability of ASCAPE-HS in a diverse community sample.

**Methods:**

The ASCAPE-HS prototype was developed through an iterative process involving researchers, clinicians, software developers, and end users under a digital health equity framework. The prototype included a self-administered, audio-delivered Frailty Index, alongside other items relevant to aging. The design process prioritized accessibility, sociocultural relevance, and technical feasibility, with regular multidisciplinary consultation and iterative refinement. Exploratory user testing with 20 older adults (aged 47‐93 years, including n=5 who had not finished secondary schooling, n=3 people with previous imprisonment history, and n=9 with mild or moderate cognitive impairment) provided feedback on usability and acceptability.

**Results:**

A 50-item Frailty Index was developed, alongside an additional selection of holistic questions that could meaningfully capture age-related health, and transferred to an iOS app (Apple, Inc), with ACASI features. Key elements included lay wording, consistent interface, simple “tapping” response options with repeatable audio feedback, a tutorial, and artificial intelligence–generated audio guidance. Key development considerations were synthesized into a checklist for teams undertaking similar projects. Successful strategies for the collaborative design process included diverse teams abreast of emerging literature and policy with varying expectations for engagement during development, and dedicating time to flexible, iterative development processes. Acceptability (median scores ≥4 out of 5 across 6 constructs) and usability (mean System Usability Scale score 79.0, SD 8.8) were high.

**Conclusions:**

A collaborative approach can produce ACASI-based health screening tools that are well-received by older adults. We highlight the feasibility of integrating frailty and aging assessment into a usable and acceptable digital tool, and offer actionable principles for collaborative, evidence-based development of equitable health screening tools in diverse, hard-to-reach populations.

## Introduction

### Digital Health Screening and Equity Challenges

Digital health screening is increasingly recognized as a promising strategy to improve the reach, access, efficiency, cost-effectiveness, and quality of health assessment for older adults, particularly in the context of rising multimorbidity and health system pressures [1,2]. However, the benefits of digital health technologies are not yet equitably distributed [3]. Older adults who are marginalized, whether due to low education or literacy, cognitive or physical impairment, cultural background, or other sociocultural exclusion, can face barriers to both traditional and digital health screening. These barriers are likely shaped by social and structural determinants of health inequity, such as limited access to technology, financial disadvantage, power imbalances, unique or restricted living environments, and complex or disrupted relationships with health care [4,5]. Moreover, digital health screening tools can assume a level of digital and written language literacy that does not necessarily reflect the lived experience of all older adults. Resultantly, those most in need of early detection and intervention may be underserved by current digital health innovations. For these groups, digital health solutions risk reinforcing existing inequities unless they are specifically developed to address these realities.

Realizing the full potential of digital health screening for such populations requires attention to “digital health equity,” defined in a recent scoping review as “a multi-level socio-ecological concept that results from fair and just opportunities for everyone to attain their highest level of health through access to technology-enabled health resources and services.” [6]. As of yet, frameworks and definitions of digital health equity are disparate, though they encompass a few common concepts, including “Equity,” “Precursor to Action,” “Action,” “Solution,” and “Result” with influential ones often traversing multiple levels of the socioecological model. For example in their Digital Health Equity Framework, Richardson and colleagues [7] detail how marginalized populations face disadvantage across multiple “digital determinants of health,” which are conditions in the digital environment that affect health outcomes at multiple levels: individual (digital literacy, technology access, and trust), interpersonal (implicit tech bias and patient-technology-clinician relationships), community (broadband infrastructure and health care digital capacity), and societal (technology policy, design standards, and algorithmic bias) levels. These digital determinants intersect with social determinants of health to create structural barriers to screening and care. Such frameworks provide useful guidance for developing and implementing equitable digital health solutions for marginalized groups.

### Audio Computer-Assisted Self-Interview Technology for Marginalized Groups

In terms of specific technologies, audio computer-assisted self-interview (ACASI) technology offers a promising, evidence-based approach to overcoming some of the barriers to digital health equity. ACASI enables self-administration of health surveys using audio prompts and simple, intuitive interfaces, reducing dependence on literacy, vision, or prior digital experience [8]. Research demonstrates that ACASI can support privacy, autonomy, and engagement, and is particularly effective in settings where stigma, low literacy, or mistrust may otherwise limit participation (refer to study by Brown et al [8] for review), while being equally useful for mainstream primary care settings [9]. ACASI has also been shown to be suitable for adults with a range of developmental disabilities, including intellectual disability and cognitive impairment [10].

ACASI embodies several human-computer interaction principles that are particularly salient for marginalized populations. For example, ACASI reduces cognitive load by leveraging dual-channel processing (visual text paired with audio), improving comprehension for users with low literacy or cognitive barriers. Importantly, ACASI operationalizes trust and privacy by removing human-to-human judgment, increasing perceived anonymity, and reducing social desirability bias. This further aligns with the CASA (Computers Are Social Actors) paradigm, where users treat the computer as a neutral, safe “interviewer.” Despite such equity-enabling features, ACASI remains underused in health screening for older, marginalized adults.

### Frailty Screening in Older Adults

In the context of health screening for aging populations, frailty is a key geriatric syndrome characterized by increasing vulnerability to stressors that is predictive of adverse outcomes and high health care use in older adults [11-14]. Early detection and better management of frailty is a clinical and public health priority [15], but many frailty measures require in-person assessment or are unsuited for self-administration by individuals with cognitive, sensory, or literacy limitations. Web-based frailty assessment tools have emerged as a potential enabler of remote frailty assessment, but consideration for accessibility and suitability for adults with limited digital access or literacy is lacking [16,17]. Similarly, while there is a growing literature on generating electronic frailty scores from existing administrative health data [18], these administrative systems and records are as yet subject to high inconsistency, as acknowledged in both Australian and international studies [19]. Moreover, for adults in secure settings or who already face difficulties accessing health care in the community, these records may be incomplete or disrupted.

Of the existing methods of measuring frailty, the Rockwood frailty index (“Frailty Index,” herein), based on the deficit accumulation model, provides a robust, multidimensional assessment that can be delivered and scored using self-report or observer-report surveys [20]. The Frailty Index is not a prescribed list of items, it allows researchers and clinicians to build their own index by selecting a number of health-related items according to a set of criteria (eg, variables that increase with age, are neither too rare nor too common, and span multiple domains), then recoding and counting the number of deficits present [21]. This structure lends itself to formats such as ACASI, as all required information can be self-reported, in a range of environments, with low resourcing requirements. Such compatibility is an opportunity for extending frailty screening to populations who struggle to access traditional, clinician-administered assessments, including those in restrictive or marginalized settings.

### Collaborative Development of Digital Health Tools

Importantly, the successful development of evidence-based tools that work in this space requires partnership between researchers, clinicians, software developers, and end users. Emerging literature has highlighted the value of engaging older adults and end users in the co-design of digital health tools, describing practical frameworks and clear benefits for intervention quality, usability, and acceptability [22,23]. However, most studies have centered on user involvement and clinician collaboration, while systematic accounts of integrated, multidisciplinary partnerships including researchers, software developers, and government stakeholders are rare. While literature provides practical models for end user engagement, much less is known about the complex process of translating research objectives, clinical expertise, and lived experience into technical specifications within software development teams. Collaborative processes between researchers and software developers where health knowledge is effectively communicated, interpreted, and realized in digital tools remain sparsely documented.

### The ASCAPE Project Context

Bringing these threads together, this study reports on the collaborative development and user testing of the ASCAPE-HS prototype, a tablet-based, ACASI-delivered screening tool interoperable with both the Frailty Index and dementia and major noncommunicable disease risk scoring. The ASCAPE-HS was developed as part of the ASCAPE (Audio App-Delivered Screening for Cognition and Age-Related Health in Prisoners) project, which aims to develop and test new digital tools to improve equitable screening of cognition, health, and functioning of older people in Australian prisons.

ASCAPE responds to the growing “aging epidemic” in prison populations worldwide, where up to 1 in 4 adults are now considered “older”(≥50 y; ≥45 for Aboriginal and Torres Strait Islander peoples) [24,25]. This population faces intersecting disadvantages: social and structural determinants predating incarceration, elevated physical and mental comorbidities, accelerated aging (exhibiting health conditions 10‐15 y earlier than community peers), and systemic health care access barriers [26-30]. While older prisoners are a minority, the barriers they face (social stigma, low literacy, disrupted health care relationships, and restricted environments) mirror challenges facing all marginalized older adults, making prisons a unique and generalizable context for testing equity-oriented digital innovations.

### Aims

This paper aims to advance the literature regarding collaborative development of equitable, evidence-based digital health screening tools for underserved, marginalized older adults by (1) presenting the steps undertaken in the development process for a self-administered, audio app-delivered Frailty Index and aging screener for older justice-involved adults; (2) describing the resultant prototype; (3) exploring usability, acceptability, and user feedback regarding the prototype; (4) synthesizing key lessons from the development process and user feedback for researchers and clinicians to consider in future work; and (5) articulating transferable principles for equity-oriented digital health tool development.

## Methods

### Ethical Considerations

Ethics approval for this research was obtained from the University of New South Wales Human Research Ethics Committee (approval number iRECS2549), the Justice Health and Forensic Mental Health Network of NSW (approval number 2021/ETH11114), Corrective Services NSW (approval number D201014950), and the Aboriginal Health and Medical Research Council of Australia (approval number 1873/21). Written consent was taken at the commencement of testing sessions with participants. To protect participant privacy and confidentiality, all data collected during user testing were deidentified at the point of collection. Participant responses were stored locally on the study iPad devices as CSV files and subsequently transferred to a secure, password-protected online folder accessible only to authorized members of the research team. No personally identifying information was linked to participant responses in the stored data files. Participants in Phase 2 user testing received Aus $100 (equivalent US $72) compensation, provided as either cash or a grocery store gift card of equivalent value, at the end of their testing session. This compensation was provided to acknowledge participants’ time and contribution to the research.

### Context: The ASCAPE Project

This work was undertaken as part of the ASCAPE study, consisting of 3 phases. Phase 1 involved co-design and consultation focus groups conducted with older adults in Australian prisons (n=20), as well as prison nurses and psychologists (n=13) to understand current health screening processes for older people, and considerations for designing digital health screening tools using serious games and ACASI technology. These informed the design and development of 2 separate iOS-based app prototypes: the ASCAPE-C, a serious game–based cognitive assessment tool, and ASCAPE-HS, a frailty-based health screener using ACASI technology. Phase 2 involved user and bug testing of the prototype apps with a sample of older adults in the community. Phase 3 (planned commencement in 2026) will involve focused usability, acceptability, and validation of the developed apps against traditional screening methods, with a sample of older justice-involved adults in Australia.

Detailed methodology and findings from Phase 1 have been published elsewhere, including specific design preferences of older incarcerated adults regarding serious game–based cognitive assessments, which informed “ASCAPE-C” app development (Arludo Inc) [31] and identified factors that are likely to impact future adoption of digital health screening tools for older people in prison [32]. This paper focuses on the development and design process for the “ASCAPE-HS” aging and frailty ACASI screener, which drew on findings from Phase 1, and presents user testing and feedback from Phase 2. These inform important directions for tool refinement for the full validation study in Phase 3.

The ASCAPE Project draws on the Framework for Digital Health Equity [7]. This framework expands the leading health disparities framework by incorporating a “digital environment” domain detailing digital determinants of health at individual, interpersonal, community, and societal levels. This work operationalized these determinants across the research process. For example, individual-level determinants (digital literacy and trust) are addressed through audio delivery and simple navigation; community-level determinants (infrastructure barriers) through offline capability; and societal-level determinants (design standards and equity norms) through strengths-based wording and culturally safe development processes. It also informs aspects such as ASCAPE’s multistakeholder governance structure and consultation processes.

### Team and Governance

All phases of this project were overseen by a collaborative, diverse research team and governance structure that was constructed to draw on all relevant technical, practical, clinical, cultural, and research expertise necessary for the project. The central team of chief and associate investigators and research staff met regularly to engage in project management, tool development, and key decision-making. This researcher-focused group consisted of leaders in frailty, prisoner health, Aboriginal health, epidemiology, neuropsychiatry and neuropsychology, dementia, and aging in marginalized populations. The software development company was in close organizational proximity to the administering university of the core research team, enabling smooth communication and as-needed inclusion within core research team meetings. A broader group of associate investigators included local jurisdiction prison health and correctional system stakeholders, a forensic psychogeriatrician, and experts in Aboriginal health assessment, who were contacted for consultation on specific elements of the study as they arose, kept updated on the broad study progress and invited to coauthor outputs where appropriate. The project was also overseen by an Aboriginal Reference group which provided cultural oversight at each phase of the study. During the project, additional ad hoc consultation was sought from a broader network of individuals with professional and research expertise in frailty and prison nursing for older adults.

### Tool Development Process and Content Development (Frailty Index and Other Questions)

The ASCAPE-HS prototype was developed via an iterative process over approximately 12 months.

Following Phase 1, a structured consultation session was held with the core research team. The aim of the session was to refine a “wish list” of domains and indicators for inclusion in the tool, as well as core design features. This list was based on a review of relevant findings and gaps in current understandings of age-related health and frailty in older, marginalized populations, and aligned with the original project grant proposal. While a key focus was to generate a Frailty Index for the tool, the discussion was kept broad to allow for inclusion of other questions or measures that would give holistic and useful information about age-related health, functioning and decline for older adults who may be subject to social and structural determinants of ill health.

During the session, the team was first given a brief recap of the study aims, the process for creating a Frailty Index [21], and the core features of ACASI technology. Then, the group discussed which core domains and indicators should be included in this Frailty Index and in the tool more broadly, focusing on findings from Phase 1 consultations, research and clinical utility, feasibility, and appropriateness for self-administration to individuals in prison. A human-centered design thinking approach guided the discussion of the core design features of the tool, building on ACASI principles and an understanding of the population and setting. A representative from the software development company for the tool was also present for this discussion, to respond to questions and ideas in terms of feasibility for development.

The first author took notes throughout the session, which were subsequently converted into a Microsoft Excel spreadsheet of items and accompanying notes regarding the development features. Through fortnightly online team meetings, ongoing review of emerging literature, and collaboration through shared documents, the questions and wording for items were finalized, and a subsequent scoring system for the Frailty Index items was also developed.

Item selection followed the standard criteria for deficit-accumulation indices described by Rockwood, with additional constraints for marginalized populations, such as self-reportability without clinical examination or medical records, and contextual adaptation for institutional settings (eg, instrumental activities of daily living items instructed participants to answer “as if living in the community”). One key trade-off was to balance Frailty Index comprehensiveness (more than 40 items recommended) within reasonable self-completion time for individuals with varying literacy and cognitive capacity. With the guidance of frailty experts, items were chosen to represent comorbidities, functioning, sensorium, self-rated health, and mood. Clinical examination items (eg, grip strength and gait speed) and record-dependent items were excluded due to access barriers in justice settings.

Only a subset of questions was selected for inclusion in the ASCAPE-HS prototype and for Phase 2 user testing. This decision was made to keep user testing sessions within a manageable timeframe, allowing sufficient flexibility for iterative refinement of both the questions and interface, and to remain within project budget constraints. Approximately 100 questions were selected to ensure representation of all question types, including multiple choice, rating scales, and numeric entry. Items anticipated to be more complex, sensitive, or likely to cause user confusion (such as incontinence and alcohol use) were also selected for inclusion to provide early important user feedback. All proposed Frailty Index items were included in the prototype.

### Software Development

Translation of the content and key functions to the prototype tool was also an iterative, collaborative process between the research team and software developers. A contract was developed between the research team and developers, denoting plans and scope for development, intellectual property, management of ongoing updates and support, data management and security, and other related arrangements. Expectations regarding development feedback and expected timelines were managed through involvement of the software developer representative in broader research team meetings and email communication.

The list of potential questions and main design features was provided to the developers, who prepared a beta version of the tool for review by the research team. Over several months, mainly through emails and online meetings, the research team and developers progressively exchanged information and feedback to refine this prototype. This process was managed mostly by a smaller development group within the research team, who communicated major updates or more important conceptual questions regarding this process to the broader team via online meetings and email. The main steps in this process included first clarifying the research team’s vision for the overall “look and feel” of the tool, then building the main functions (eg, volume control and color scheme) and response options (ie, how the user enters their responses and navigates through the survey), then finalizing question text as it appears on screen and via audio, and selecting specific audio and visual content.

Simplicity, consistency, and clarity were prioritized for visual design suitable for older adults based on relevant evidence [33,34]. A blue and white color scheme was chosen to address common color vision deficiencies. A sans-serif font was chosen for superior legibility for older adults. Further, the iPad mini (Apple, Inc) was chosen for its optimal balance between screen size and device weight, maximizing readability without compromising device usability.

All text, audio, and visual content for each question and response option was sourced and prepared by the research team and shared with the software developers to apply to the app. Images were icons selected by the researchers from an online repository licensed by the research team’s administering institution. These icons were chosen to be clear, simple, and universally recognizable. Ambiguous images (eg, “skip button”) were discussed within the core research team. For audio, the use of a professional voice actor or member of the research team was considered and trialed. However, it was ultimately decided that the use of an artificial intelligence (AI) voice would allow consistency and flexibility for future development of the tool. The audio was generated using a paid, online AI text-to-speech conversion program. A number of different voices were trialed and the final decision was made by the research team consensus. A specific female voice with an Australian accent was chosen, informed by end user needs for clarity of voice, relatively mild accent and calm delivery. The research team uploaded the script, then downloaded and separated the audio into individual clips. Manual editing of the audio was required to ensure breaks, tone, accent and speed were suitable.

A shared online document and online folder were used to share all the selected icons, written and audio content, and a naming convention was agreed upon for each of the files. The document was used by researchers to systematically denote precise text, audio, and visual files to apply to each screen, the proposed layout of the response options (eg, horizontally under the question or to the side), and how users can enter their responses (eg, tap response button or enter number into keypad). This system streamlined the development process and thereby reduced costs; for example, any changes made by the research team could then easily be turned into relevant (JSON) files, which could be uploaded to the app without any additional steps by the development team. Data were designed to be saved locally on the administering device without the need for internet connectivity—a unique consideration for secure settings.

During this process, 2 “playable” iterations of the app were developed and circulated to the broad research team for initial bug testing and feedback. As part of this process, the research team reached out to relevant individuals within their broader networks for initial feedback, such as older friends and family members, forensic and custodial health professionals, sand researchers. Feedback was collated and incorporated by the developers before the prototype was deemed ready for user testing with a community sample (Phase 2).

The research team retained full ownership and control over all aspects of study design, data collection, analysis, and interpretation. Access to participant data was restricted to named research team members only; the software development company had no access to raw participant data at any stage of the study. All data analysis and interpretation were conducted independently by research team members without developer involvement. These governance arrangements were formalized in the research ethics protocols, ensuring clear boundaries between technical development and research independence.

### Prototype Feedback and User Testing

User testing sessions were designed for feedback on the acceptability and usability of the tools (both ASCAPE-HS and the ASCAPE-C) and to identify any issues or bugs in the products, with a community-based sample of older adults. This would enable further refinement of the prototypes for full validation and user testing with an older justice-involved sample in Phase 3. Acceptability refers to how appropriate or suitable an intervention is perceived to be by those who use or deliver it, based on their cognitive and emotional responses to it [35]. Usability is commonly defined as “the extent to which specified users can use a system or tool in a specified context to achieve specified goals with effectiveness, efficiency, and satisfaction” [36]. Usability thus captures users’ perceptions of the simplicity, ease of use, and learnability of a digital tool, as well as their confidence and satisfaction when using the tool.

Inclusion criteria included being 50 years or older or 45 years or older if an Aboriginal or Torres Strait Islander person, as aligned with the definition of an “older person” in Australian prisons. Participants who did not have sufficient English to self-complete and give feedback about the apps were excluded. Those diagnosed with dementia were also excluded from this phase. Participants were recruited via physical and online study flyers circulated through a diverse range of organizations and groups within the Sydney region, in New South Wales, Australia, including homelessness service providers, postrelease transition service providers, local community groups targeting older people, Aboriginal research programs, and forensic and general allied health clinics. Snowball sampling was used alongside these recruitment pathways. These were selected to gain a diverse sample in terms of age, gender, education level, cultural background, and socioeconomic status to test the apps, though participants were not formally screened for these attributes. Participants expressed interest by contacting the research team via email or phone, were screened for inclusion, and sent copies of the participant information statement and consent form to read and complete. All individuals who expressed interest and contacted the research team (n=20) met eligibility criteria, enrolled, and completed the full testing session (100% completion rate).

User testing took up to 2 hours per person (including a break) and was conducted in person by 2 administrators, in a private room either at the research team’s university campus or at a local homelessness service provider. User testing involved 2 sessions separated by 1 break. The first, ASCAPE-C focused session involved (1) a visual acuity test, (2) administration of the Montreal Cognitive Assessment (MoCA) [37], (3) completion of the ASCAPE-C on an iPad mini, (4) semistructured qualitative feedback interview on user experience (audio-recorded), and (5) completion of a 16-item user experience and usability scale comparing the MoCA to the ASCAPE-C developed by the research team. Participants then had the option of a brief break, followed by the ASCAPE-HS session, which involved (6) self-completion of the ASCAPE-HS wearing over-ear noise-canceling headphones and using an iPad mini; (7) audio-recorded feedback interview consisting of a mix of brief, open-ended, and Likert-style questions where participants rated the tool on 6 theoretical constructs of acceptability (affective attitude, burden, ethicality, opportunity costs, perceived effectiveness, and self-efficacy) [35]; (8) a few additional open-ended questions prompting feedback for tool improvements; and (9) completion of the 10-item System Usability Scale (SUS [38]), either self-administered using pen and paper, or read to participants and filled in on their behalf by the administrator. The SUS has been deemed suitable for evaluating usability in digital health apps [39].

This paper presents the ASCAPE-HS feedback. Data from the screener were transferred from the device to an online, secure shared folder accessible to the research team, alongside participant responses to the Likert scales regarding acceptability and usability of the tool. Analysis was undertaken by the first author and included producing descriptive summaries of participant demographics, MoCA scores (reviewed for scoring consistency and accuracy by the senior author), computation of the Frailty Index, and description of participants’ answers to the Likert scales regarding acceptability and usability (SUS) using SPSS statistical software (IBM Corp). There was no missing data for ASCAPE-HS screener items, acceptability ratings, or SUS responses.

Open-ended questions inviting feedback on 5 aspects of the tool were transcribed and subject to deductive content analysis. This descriptive analysis aimed to elicit actionable user feedback for tool refinement rather than generate interpretative qualitative findings. It involved listening to audio recordings and reading transcripts multiple times to gain familiarity with the data. Responses were identified at the semantic level (what was directly said by participants, with minimal interpretation) and coded for content corresponding to the 5 feedback areas (tutorial, audio, visual design, response options, and overall experience). The first author coded all transcripts, and a second researcher also coded a subset of 5 interviews separately. Discrepancies were discussed until consensus was reached. Similar responses were grouped into categories based on commonalities, and illustrative quotes were selected for the paper.

## Results

### Functionality and Key Design Features

**Figure 1.**
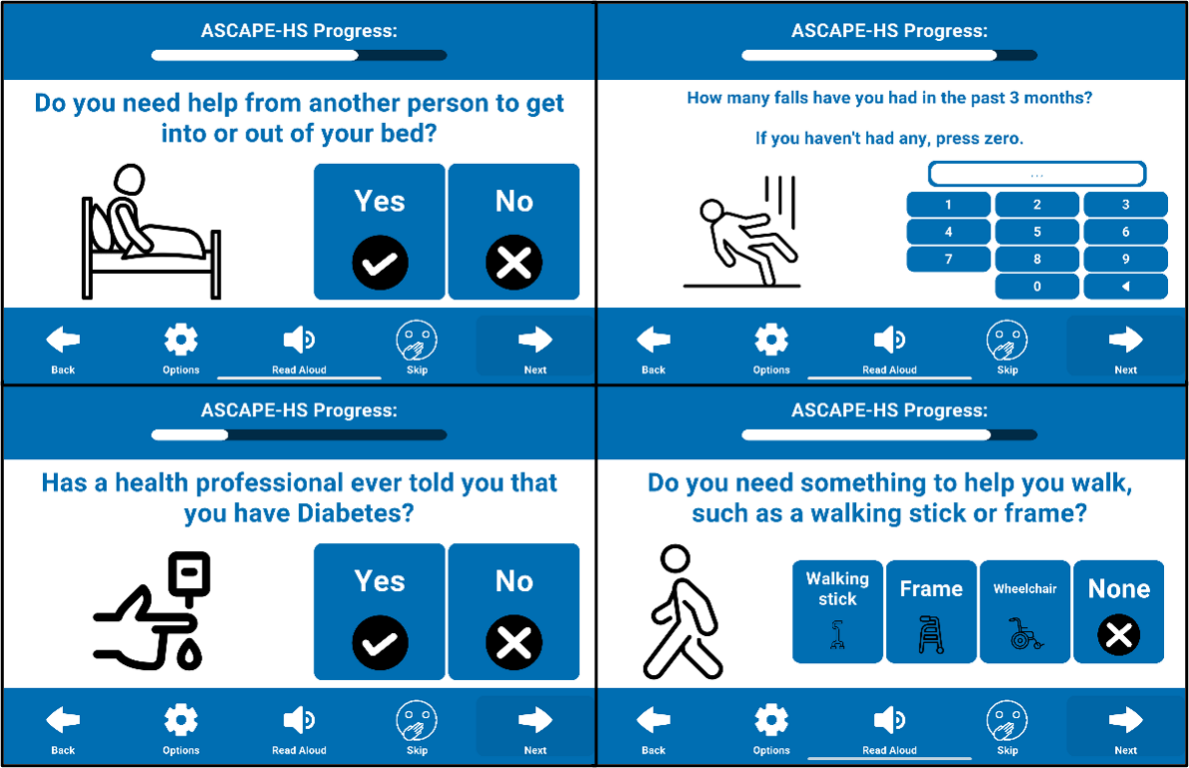
Selection of screenshots from ASCAPE-HS (Audio App-Delivered Screening for Cognition and Age-Related Health in Prisoners) prototype app.

Figure 1 displays a selection of screens from the ASCAPE-HS prototype.

Key aspects of its interface and functionality included:

1. Delivered on an Apple iPad mini (6th gen); 8.3-inch or 21.08 cm screen diagonally; 293 g
2. Bluetooth-enabled, noise-cancelling, over-ear headphones were provided for participants.
3. An interactive tutorial guided participants through the toolbar functions and provided opportunities to practice responding to questions using the touchscreen; the tutorial could be repeated as often as needed before beginning the main screener.
4. A fixed toolbar was positioned at the bottom of the screen, featuring volume adjustment, skip, next, and back navigation, and a “read aloud” icon to replay audio for questions and instructions.
5. All question screens shared a consistent layout, with a progress bar displayed at the top, the toolbar at the bottom, and primary content (question and response options) occupying the central area.
6. A cohesive blue and white color scheme was applied throughout. Responses were highlighted in red if selected.
7. All questions and possible responses were accompanied by clear image icons to aid universal comprehension and engagement. All icons were simple black outlines (as opposed to photos or more complex illustrations).
8. Audio instructions played automatically for each question screen, and audio feedback was available for every interactive button on the screen, including the toolbar and response options for questions.
9. Responses were submitted by tapping the option, followed by tapping “Next”(or “Skip/Prefer not to say”) to proceed. Participants could tap any of the responses repeatedly, and repeat the audio instructions as often as needed, before submitting responses via the “Next” or “Skip” buttons.
10. The resultant data were stored locally on the iPad as a CSV file.

### Frailty Index

Fifty indicators were chosen to form the Frailty Index (Table 1). The index included n=2 items measuring global self-rated health and health decline, n=17 comorbidities, n=3 indicators of sensorium, n=23 indicators of functioning, and n=5 indicators of mood.

**Table 1.** Number and domain of Frailty Index items (N=50).

Domain (number)	Indicators
Self-rated health (n=2)	Self-rated health; self-rated health decline in the past year
Comorbidities (n=17)	Blood pressure; chronic lung disease; stroke; heart attack; diabetes; cancer; osteoporosis; arthritis; high cholesterol; kidney disease; liver disease; macular degeneration; cataracts; periodontitis; dementia or Alzheimer; polypharmacy; excess alcohol use
Functioning (n=23)	Mastication; oral pain; appetite; weight loss; tiredness; trouble sleeping; activities of daily living; instrumental activities of daily living; mobility; balance; falls; incontinence; memory
Sensorium (n=3)	Hearing; vision; pain
Mood (n=5)	Feeling: relaxed, thinking clearly, cheerful, optimistic, and close to other people

A full list of items and their scoring system for the Frailty Index is also available in Multimedia Appendix 1. Most Frailty Index items outside of the “comorbidities” domain were based on existing validated measures that have been used in older populations, such as the Patient Health Questionnaire-15 [40] or the Geriatric Postal Screening Survey [41]. However, wording adaptations were made, for example, for instrumental activities of daily living, which may not be directly relevant to prison settings, participants were specifically instructed to answer as if they were living in the community. Rewording of items to be shorter or more conversational was also often necessary to avoid crowding the screen and to improve interpretability for people with low literacy. Where possible, strengths-based items were used to improve the experience of user safety, for example, items from the Warwick-Edinburgh Mental Wellbeing Scale [42] were selected to measure mood.

### Other Questions: Holistic Health, Dementia Risk Score, and Prison-Specific Items

In addition to the Frailty Index, the ASCAPE-HS prototype incorporated a range of demographic items and supplementary questions designed to provide a more comprehensive picture of health, well-being, and functioning in older adults. We also incorporated a small number of items tailored for justice-involved older adults (eg, history of severe mental illness and head injury). During the course of development, the dementia and other noncommunicable diseases (DemNCD) risk assessment score was published [43]. The DemNCD score, like a Frailty Index, combines multiple factors obtainable via survey or existing health data, into a single measure that determines an individual’s risk of developing dementia, stroke, heart attack (myocardial infarction), and diabetes, Notably, there was considerable overlap between factors used in the DemNCD and those already included in both the Frailty Index and the intended screener overall, and thus inclusion of all DemNCD factors would allow both scores to be generated from the same dataset with minimal additional questions, maximizing utility of the tool. Due to development restraints, only a subset of additional DemNCD items were included in the ASCAPE-HS prototype; all items will be included in the final tool for Phase 3 testing.

### Usability, Acceptability, and Suggestions for Improvement

#### Sample Demographics

Twenty older adults from the community participated in user testing of the ASCAPE-HS prototype (May-September 2025). Participant demographics, MoCA scores, and Frailty Index scores are presented in Table 2. Administrators observed approximate completion times of 15‐20 minutes across with no navigation errors or requests for help noted. Data are almost complete, except for self-reported height (7 missing values) and weight (4 missing values). One affirmed having a diagnosis of intellectual or learning disability, but then skipped the follow-up question regarding its severity, likely indicating uncertainty.

**Table 2.** Demographic characteristics of user testing participants (N=20).

Characteristic	Value
Age (y), mean (SD; range)	65.5 (14.3; 47‐93)
Male, n (%)	13 (65)
Aboriginal, n (%)	2 (10)
Self-rated spoken English level, n (%)	
Very well	16 (80)
Well	4 (20)
Finished high school, n (%)	
Yes	15 (75)
No	5 (25)
Ever told you had a learning disability or intellectual disability?, n (%)	
Yes	3 (15)
No	17 (85)
Cognitive impairment (MoCA^a^), n (%)	
No impairment	11 (55)
Mild impairment	8 (40)
Moderate impairment	1 (5)
Frailty Index, mean (SD; range)	0.16 (0.12; 0.01‐0.4)
Frailty Index category, n (%)	
Not frail (<0.08)	5 (25)
Prefrail (.08-.24)	9 (45)
Frail (≥0.25)	6 (30)

a MoCA: Montreal Cognitive Assessment.

#### Acceptability

Participants’ ratings on 8 Likert-style items measuring acceptability are presented in Table 3. Across all acceptability constructs, most participants gave positive ratings and feedback, with median scores anchored strongly toward positive responses for each item. However, at least one individual typically reported difficulties or challenges for each item.

**Table 3.** Scores for acceptability by item and acceptability construct (mean, median, and IQR).^a^

Item (rating)	Acceptability construct	Participants, n	Mean (SD)	Median (IQR)
How much did you like it? (1=strongly dislike, 5=strongly like)	Affective attitude	20	4.25 (0.716)	4.0 (1)
How much effort did it take? (1=no effort at all, 5=huge effort)	Burden	20	1.50 (0.513)	1.0 (1)
How confident would you be to use it by yourself? (1=very unconfident, 5=very confident)	Self-efficacy	20	4.65 (0.933)	5.0 (0)
Could you rate how unkind or unfair it may be? (1=very fair or kind, 5=very unfair or unkind)	Ethicality	20	1.70 (1.031)	1.0 (1)
How strongly do you agree that it could help improve health care for older people? (1=strongly disagree, 5=strongly agree)	Perceived effectiveness	19	4.53 (0.612)	5.0 (1)
How strongly do you agree that it is worth people’s time to do this to improve their health care? (1=strongly disagree, 5=strongly agree)	Opportunity costs	19	4.68 (0.582)	5.0 (1)
How strongly do you agree that it could help improve health care for older people in prison? (1=strongly disagree, 5=strongly agree)	Perceived effectiveness	3	4.67 (0.577)	5.0 (NA^b^)
How strongly do you agree that it is worth people’s time in prison to do this to improve their health care? (1=strongly disagree, 5=strongly agree)	Opportunity costs	3	5.00 (0.00)	5.0 (0)

a Items relating to the acceptability of the tool in prison were only asked of those with lived experience.

b Not available.

#### Usability

Participants’ ratings for the SUS are presented in Table 4. The aggregate mean usability score for the sample overall was 79.0 (SD 8.8; range 50‐90), indicating good overall endorsement of usability [44], with especially high scores in terms of simplicity, integration of features, confidence, and ease of use. The lowest scores for usability were for frequent use of the tool (item 1) and belief that most people would learn to use the tool quickly (item 4).

**Table 4.** System usability scale scores by item (mean, median, and IQR).

Scale item (1=strongly disagree, 5=strongly agree)	Mean (SD)	Median (IQR)
I think that I would like to use this tool frequently	3.75 (1.251)	4.0 (2)
I found the tool unnecessarily complex	1.10 (0.308)	1.0 (0)
I thought the tool was easy to use	4.65 (0.745)	5.0 (1)
I think that I would need the support of a technical person to use this tool	1.55 (1.146)	1.0 (1)
I found the different features of the tool worked well together	4.75 (0.444)	5.0 (1)
I thought there was too much inconsistency in this tool	1.50 (0.889)	1.0 (1)
I would imagine that most people would learn to use this tool very quickly	4.05 (0.759)	4.0 (2)
The tool was smooth and straightforward to use	4.65 (0.489)	5.0 (1)
I felt very confident using the tool	4.70 (0.470)	5.0 (1)
I needed to learn a lot of things before I could get going with this tool	1.50 (0.889)	1.0 (1)

#### Prompted Feedback: Audio, Breaks, Ordering, Interaction Mode, and Other Suggestions

Participants were prompted for specific feedback on 5 aspects of the tool (voice, breaks, other ways of answering questions, question ordering, and other suggestions).

1. Voice preferences:
  Most found the digital tool’s voice (female, neutral accent; 18/20) acceptable or preferable, while there were no strong preferences for gender, some female participants preferred a female voice (“I think I prefer the female voice.” [P3]; “I thought the female voice was excellent…It’s just nice. It’s sort of neutral and non-challenging, you know...” [P10]).
2. Session breaks:
  Nearly all (19/20) preferred completing the screener in a single sitting, saying a break was unnecessary (“Yeah, I’d rather do it straight,” [P1]). Only one mentioned that breaks might help some users, suggesting this should be an option for those with fatigue or concentration issues. (“If you’ve got concentration problems, it would be difficult to sort of keep at it.” [P10])
3. Question ordering:
  The majority found the question order logical and acceptable. A few valued or suggested the option to skip, return, or flag items, especially for sensitive questions. (“Maybe allow the skip and then at the end go back and allow those questions.” [P3]). No one identified a need for reordering.
4. Interaction mode:
  Most participants strongly preferred tapping and simple interfaces (16/20), with a few requesting additional modes such as spoken response, sliding scales, or free text for greater nuance. Simplicity, clarity, and accessibility for arthritis or low digital literacy were cited as important. (“It could be hard for someone with difficult abilities, I should imagine, if they don’t understand technology at all. Yeah. But otherwise it’s quite straightforward.” [ P12]; “I suppose with increasingly with, with technology moving, you know, into the Siri (voice-activated) types. that people are used to. Then, yeah, that could be the way to go.” [P9])
5. Other suggestions made by multiple participants included:
  - More flexible response options, that is, both the option to respond as being “unsure” to a question, or the ability to flag or clarify questions and add additional detail (eg, free-text entry) for the health professional to review alongside the person’s response.
  - A number of once-off suggestions also included: consistent text size for response buttons, a larger screen, and more standardization of visual icons. (“It could be tidier with just this, like a bit more standardized in the style of pictures and so on.” [ P19])

## Discussion

### Overview of Findings

These findings offer novel insights into the use of ACASI technology to measure aging, the development of an equitable and digitally transferable Frailty Index, and considerations for digital tool development within highly multidisciplinary groups. Our findings suggest ASCAPE-HS achieves the dual goal of being functionally effective (usability) and contextually appropriate for marginalized older adults (acceptability), supporting readiness for Phase 3 validation with justice-involved populations. High usability (mean SUS 79.0, SD 8.8, “good” range) indicates the tool’s interface, navigation, and interaction design function effectively for older adults with diverse literacy and cognitive abilities. High acceptability across 6 constructs (median ≥4/5; IQR 0-1) indicates the tool is perceived as appropriate, nonburdensome, and ethically suitable for the target population. Critically, both are necessary: high usability with low acceptability would suggest a technically sound tool that users reject as inappropriate; high acceptability with low usability would suggest conceptual appropriateness undermined by poor implementation. Overall, the 15‐ to 20-minute completion times, zero noted navigation errors or requests for help, minimal skipped questions (height and weight uncertainty; 1 disability severity), and high data completeness are highly positive observations.

### Methodological Contribution

#### Overview

The study makes an important methodological contribution to equity-oriented digital health development. Kim and Backonja [6] scoping review identified that existing digital health equity frameworks often lack actionable guidance for how to implement equity-focused design processes. Our documentation of multistakeholder governance and consultation processes, as well as the evidence-to-software translation workflows (file systems and iterative beta testing), has yielded transferable insights. These lessons have been operationalized into an actionable development and user testing checklist (Multimedia Appendix 2), providing structured guidance for collaborative development of accessible digital health screening tools in marginalized populations and resource-constrained environments. Table 5 provides a condensed version of this checklist.

**Table 5.** Condensed checklist for collaborative development of equitable ACASI^a^-based digital health tools

Domain	Checklist items
Multistakeholder governance	Assemble diverse core team (clinical/epidemiological/technical/cultural); create development subgroup for daily decisions Engage end users early (clinicians/administrators/target population); establish advisory/reference groups for cultural oversight Regular meetings with phase-appropriate engagement; communicate updates across broader team
Software development partnership	Select proximate development partner; establish formal contracts (scope/IP/data/security/support) Include developer representative in core meetings; budget adequate time/resources for iterative cycles Create shared documentation systems (folders/naming conventions) for streamlined specifications
Regulatory and ethical	Obtain ethics approvals from all relevant jurisdictions Develop signed team protocol
Evidence-informed content development	Conduct co-design sessions with target populations Ongoing review of relevant literature Ground project in digital health equity framework Hold structured core team “wish list” sessions for domains/features; seek ad hoc expert consultation Document rationale for inclusion/exclusion decisions
Iterative refinement process	Budget sufficient time for multiple iteration cycles Conduct internal team testing plus informal feedback before formal testing Create systematic documentation tracking items/wording/scoring changes
Accessibility and universal design	Implement audio narration for all items; consistent color scheme/fonts/contrast; universal icons Select balanced device (screen size/weight); provide noise-cancelling headphones Include interactive repeatable tutorial; test visual content for cross-cultural appropriateness
Interaction design features	Use tap-only responses; consistent layout (fixed toolbar/progress bar/predictable structure) Provide navigation controls (back/skip/next); allow audio replay and flexible pacing Visual feedback (highlighted selections); audio confirmation for interactions
Content adaptation principles	Use lay language, strengths-based, nonstigmatizing wording; shorten for comprehension Make conversational adaptations while maintaining validity Adapt context-specific items for institutional settings; balance comprehensiveness with completion time
Audio development	Consider AI^b^-generated voice for flexibility; select based on end user needs (clarity/accent/pace/tone) Conduct core team script review for accuracy/sensitivity; manual editing for quality
Data management and security	Design for offline use with secure local storage; weigh privacy versus real-time access needs Recognize jurisdiction-specific requirements; plan context-specific adaptation Plan for future deployment security/privacy requirements (encryption at rest/transit, MDM^c^, and audit trails)
User testing protocol	Define inclusive criteria (age/education/culture/cognition/life experience); recruit purposively Allow adequate time/comfort; observe completion and note technical issues Conduct structured feedback (acceptability constructs, SUS^d^, and design elements)
Analysis and refinement	Analyze quantitative usability/acceptability data; conduct qualitative content analysis Test for missing data/completion issues; identify bugs requiring developer attention Document user suggestions; plan iterative refinement before validation study
Transparency and reporting	Disclose dual roles and intellectual property arrangements; clarify developer involvement in authorship Acknowledge community testing versus target setting validation limitations Outline future validation studies, implementation research, longitudinal outcomes, and population adaptations

a ACASI: audio computer-assisted self-interview.

b AI: artificial intelligence.

c MDM: Master Data Management.

d SUS: System Usability Scale.

#### Lessons for Multidisciplinary, Collaborative Development

The ASCAPE-HS development process demonstrated that effective multidisciplinary collaboration requires intentional structural supports beyond diverse team composition. Clear relationship infrastructure—built through existing working relationships, phase-appropriate meeting arrangements, and systematized communication channels—enabled rapid problem-solving and shared ownership while respecting stakeholders’ varying availability and expertise. Organizational proximity between developers and researchers fostered trust and real-time refinement, while cross-disciplinary involvement built mutual capacity, with developers gaining clinical perspective and researchers improving technical communication proficiency. Multiple low-stakes iterative feedback rounds reduced risk and validated the approach, yielding high usability (SUS score of 79.0) and acceptability (median ≥4/5; IQR 0-1), with shared platforms and versioning systems efficiently translating content into functional app features while minimizing errors and costs.

#### Lessons for Tool Design

Acceptability and usability scores indicated that participants had overall positive feelings toward the tool, agreed with its potential utility, and found it easy and nonburdensome to use. These findings confirmed that centering lived experience from project inception fundamentally shapes equitable tool development. Continual consultation with justice-involved older adults and frontline professionals ensured sociocultural relevance, with strengths-based plain language, culturally familiar AI audio, and noninfantilizing design elements achieving high ethicality ratings (median 1.0; IQR 1, “very fair or kind”). Literacy barriers were systematically reduced through comprehensive audio narration, replayable tutorials, tap-only inputs, and lay wording, enabling independent completion across education levels. Autonomy was embedded through skip options, headphone privacy, and practice tutorials, yielding high self-efficacy (median 5; IQR 0, “very confident”) and low burden (median 1; IQR 1, “no effort at all”). Universal design elements (high-contrast colors and recognizable icons) and handheld devices ensured accessibility, including for participants with cognitive impairment (n=9).

### Implications, Limitations, and Next Steps

To our knowledge, this is the first known application of ACASI technology for screening age-related health in older adults and demonstrates that the ACASI technology shows promise for self-administration of a digital health screener for a range of older people in the general community in Australia. Importantly, many elements of the ASCAPE-HS are likely to be relevant for other hard-to-reach groups, and were designed with broad transferability to other underserved or low-resource settings in mind. Core design features are applicable across contexts where literacy barriers, digital exclusion, health care disruption, or stigma limit screening access, including people experiencing homelessness, rural or remote communities, residential aged care, and low-resource primary care settings.

As an extension of existing applications of ACASI in literature, the AI-generated audio approach offers future adaptability advantages: new audio files can be cost-effectively generated for different languages, accents, or content revisions without requiring repeated professional voice actor sessions, supporting scalability across diverse populations and linguistic contexts. Potential risks arising from the use of an AI voice (synthetic comprehension bias and gender perception) were mitigated via team quality assurance processes, editing, and Phase 2 validation (no issues; SUS score: mean 79.0, SD 8.8).

However, context-specific adaptations are essential. Frailty Index content and additional health questions must be tailored to population-specific health profiles, risk factors, and clinical priorities. Cultural safety processes are transferable but require engagement with context-specific community advisors and cultural experts. Regulatory and ethical requirements vary by jurisdiction. Our offline data storage addressed correctional security restrictions, but other settings may prioritize cloud-based systems for care integration. International adaptation requires consideration of digital infrastructure availability, linguistic or cultural adaptation and health care system integration requirements. Of note, data-at-rest encryption was not implemented in the Phase 2 prototype but may be required for clinical deployment, adding device provisioning complexity that could create barriers in low-resource settings. Also, the manual transfer that occurred in this study is not scalable for routine clinical use, and automated secure transfer or health system integration would be needed.

User testing findings were based on a community sample (n=20) and should be interpreted with caution when considering application to other marginalized groups. While the sample was diverse in important ways, including age range (47‐93 y) and higher-than-expected representation of people with learning or intellectual disability and cognitive impairment compared to the general Australian population, several limitations shape interpretation. The sample size, while adequate for exploratory usability testing and identifying major interface issues, limits statistical power for detecting subgroup differences and may not capture less frequent usability problems. Convenience and snowball sampling may introduce selection bias toward individuals comfortable engaging with research or connected to service providers, potentially underrepresenting the most marginalized. Exclusion criteria, requiring English proficiency and excluding diagnosed dementia, limit generalizability to non–English-speaking older adults and those with advanced cognitive impairment, groups overrepresented in prisons who face the greatest digital equity barriers.

While the ASCAPE-HS design was informed by Phase 1 co-design with incarcerated participants who discussed trust, privacy, and disclosure concerns in custodial settings, direct assessment of usability and acceptability within prison environments is a critical next step in Phase 3 of the study. Implementation and scalability considerations, including resource requirements, maintenance, and health system integration, were not examined in Phase 2 user testing and will be addressed systematically in Phase 3 through consultation with prison health staff and correctional stakeholders. The prison context may significantly alter user experience in ways not captured by Phase 2 testing. Institutional mistrust, power dynamics between prisoners and correctional staff, surveillance concerns, environmental constraints (ambient noise, competing institutional demands, and limited private space), and restricted autonomy may all influence how justice-involved adults interact with digital health tools and perceive their safety and acceptability. Actual in-prison deployment during and after Phase 3 will require enhanced security protocols developed with relevant custodial and health stakeholders. Planned explorations will address key elements tailored to justice system standards and infrastructure, including encryption at rest and in transit, secure automated data transfer, device management and managed distribution, role-based access controls, and audit logging.

Of note, despite governance structures designed to promote equitable collaboration, inherent power asymmetries remained. Ultimately, the core research team retained final decision-making authority over tool content and design, reflecting pragmatic constraints rather than fully participatory co-design or equal stakeholder decision-making. Power imbalances also existed among stakeholder groups: tensions between clinical priorities, correctional security requirements, Aboriginal cultural safety principles, and research objectives were negotiated throughout development, but competing interests were not always fully reconciled. Phase 1 consultations occurred within the prison environment where participation, though voluntary and ethically approved, cannot be entirely free from institutional coercion. Budget and timeline constraints limited the depth of iterative engagement possible with end users and community representatives. These tensions between research efficiency and meaningful power-sharing, and between diverse stakeholder interests, represent ongoing challenges in digital health equity work with marginalized populations that require continued methodological innovation and critical reflexivity.

Taken together with the DemNCD risk scoring planned for development in Phase 3, the ASCAPE-HS screener represents the first known effort to provide a digital assessment capable of simultaneously generating composite frailty, dementia, and major noncommunicable disease risk indices. This is an innovation with significant potential for broader population health screening as well as research.

Another important contribution of this work is the development of an accessible, digitally self-administrable composite Frailty Index. This Frailty Index was specifically developed to meaningfully and acceptably capture age-related health in marginalized adults who may display aging at a relatively younger age, with consideration for complex health and personal histories and relationships with health care professionals and systems. It encompasses a diverse and strong number of indicators (40 or more are advised for a robust index, Searle et al [21]). Moreover, the questions are suitable for transfer to an audio-delivered, digital, self-administered format and can be self-administered in less than 20 minutes. While validation of our Frailty Index is not a focus of this study or appropriate for the sample, it is worth noting that the mean Frailty Index score (0.16; SD 0.12) was lower than that of another large Australian study, which found that Australians undergoing aged care eligibility assessment had a mean Frailty Index score of 0.20 (n=903,996; mean age 82 y). This is in the expected direction due to the significantly older age and higher care needs in this cited study. Further review and refinement of the items, validation, and user testing of the Frailty Index with adults in prison will occur in Phase 3 of the study and will be published separately.

Phase 2 user testing examined usability and acceptability only—not clinical validity, screening accuracy, sensitivity, specificity, or predictive utility. Clinical validation of ASCAPE-HS against clinician-administered frailty assessments and health outcomes is required in Phase 3 before conclusions about screening effectiveness can be drawn. Moreover, the findings about acceptability and usability have been discussed only in the context of the quantitative scores provided by participants. While scores were mainly positive, each item displayed a range of scores (eg, confidence to use the tool alone ranged from very unconfident=1 to very confident=5), implying nuance to participants’ experience that was not fully captured quantitatively. An additional in-depth exploration of the qualitative feedback provided by the user testing sample was not within the scope of this work and will be undertaken as a separate study. This will allow a more complete understanding of the “why” and “for whom” digital, ACASI-based health screening may be most suitable.

### Conclusion

These findings demonstrate how a multidisciplinary, collaborative development process can be used to develop digital health innovations for marginalized or hard-to-reach older adults in unique settings. Collaborative, co-designed digital ACASI-based solutions can overcome many of the barriers faced by older adults experiencing marginalization, offering a viable pathway for health screening in settings where traditional assessment is often not feasible.

Moving forward, implementation research examining workforce integration, resource requirements, and real-world feasibility in prison health settings is essential. Comparative evaluation of ACASI-delivered versus clinician-administered screening in terms of completion rates, disclosure quality, and clinical utility will inform optimal deployment models. Longitudinal studies tracking health outcomes and health care use following ASCAPE-HS screening can establish clinical impact. Finally, adaptation and validation in other marginalized populations (people experiencing homelessness, rural communities, and residential aged care) will test the transferability of design principles and assess cross-context effectiveness.

We encourage others to consider the actionable principles from the findings in future work with hard-to-reach groups. In particular, policymakers, funders, and health service leaders are encouraged to support development approaches that embrace diverse teams collaborating over sufficient development and testing periods, investing in adaptable and accessible technology solutions, and further supporting the evaluation of the impacts of these innovations on measurable health and equity outcomes.

## Supplementary material

10.2196/85768 Multimedia Appendix 1 Questions, responses, and Frailty Index scoring for items included in the ASCAPE-HS prototype.

10.2196/85768 Multimedia Appendix 2 Development and user testing checklist for equitable, audio computer-assisted self-interview–based digital health screening tools in low-resource settings.

## Abbreviations

ACASI: audio computer-assisted self-interview
AI: artificial intelligence
ASCAPE: Audio App-Delivered Screening for Cognition and Age-Related Health in Prisoners
CASA: Computers Are Social Actors
DemNCD: dementia and other noncommunicable diseases
MoCA: Montreal Cognitive Assessment
SUS: System Usability Scale
