# Supplementary material for: Equitable Digital Frailty Screening for Marginalized Older Adults Using Audio Computer-Assisted Self-Interview: Collaborative Development Guide and User Testing Study
Source: JMIR Form Res. 2026 May 13;10:e85768. doi: 10.2196/85768 (PMC13170664; doi:10.2196/85768)
Supplement: Multimedia Appendix 1 [file formative-v10-e85768-s001.pdf]

Multimedia Appendix 1: Questions, responses, frailty index scoring for items included in the ASCAPE-HS prototype.

	Question	Response options (Frailty Index score weighting)
1	When you were born, which sex was recorded on your birth certificate?	Male<br>Female<br>Something else
2	What is your gender now?	Male<br>Female<br>Non-binary<br>Something else
3	Were you born in Australia or overseas?	Australia<br>Overseas
4	How many years have you lived in Australia?	[Number entry]
5	How well do you speak English?	Very well<br>Well<br>Not well
6	In terms of marriage, are you…	Single and never married<br>Have a regular partner<br>Married or de facto<br>Separated<br>Divorced<br>Widowed
7	Are you an Aboriginal person, a Torres Strait Islander, both or neither?	Aboriginal<br>Torres Strait islander<br>Both<br>Neither
8	Do you know your tribe or mob?	Yes<br>No
9	Do you usually feel connected to your Aboriginal or Torres Strait Islander culture?	Yes<br>No
10	Did you finish high school or secondary school?	Yes<br>No
11	What is the highest education that you finished?*	High School or Secondary School<br>College certificate or Diploma<br>Technical or Trade qualification<br>University degree or other tertiary qualification
12	Did you finish primary school?	Yes<br>No

	Question	Response options (Frailty Index score weighting)
13	Did you finish the school certificate in Year 10, or did you leave school before then?*	Finished<br>Left before then
14	Has a professional ever told you that you have intellectual disability or a learning disability?	Yes<br>No
15	Did they tell you that the intellectual disability was mild, moderate, or severe?*	Yes- mild<br>Yes – moderate<br>Yes – severe<br>No – they didn't tell me
16	In general, would you say your health is:	Poor (1)<br>Fair (0.5)<br>Good (0)<br>Very Good (0)<br>Excellent (0)
17	Compared with 1 year ago, how would you rate your health in general now	Much better now (0)<br>Somewhat better now (0)<br>About the same (0)<br>Somewhat worse (0.5)<br>Or much worse (1)
18	How many doctor-ordered, prescription medications do you take?	[Number entry]<br>0 (0)<br>1-4 (1)<br>5-9 (2)<br>10-14 (3)<br>15-19 (4)<br>20+ (5)
19	Has a health professional ever told you that you have high blood pressure?	Yes (1)<br>No (0)
20	Has a health professional ever told you that you have a chronic lung disease, such as asthma or emphysema?	Yes (1)<br>No (0)
21	Has a health professional ever told you that you have had a stroke?	Yes (1)<br>No (0)
22	Has a health professional ever told you that you have had a heart attack?	Yes (1)<br>No (0)
23	Has a health professional ever told you that you have diabetes?	Yes (1)<br>No (0)
24	Has a health professional ever told you that you have cancer?	Yes (1)<br>No (0)
25	Has a health professional ever told you that you drink too much alcohol?	Yes<br>No
26	Has a health professional ever told you that you have osteoporosis, or a hip fracture?	Yes (1)<br>No (0)
27	Has a health professional ever told you that you have arthritis?	Yes (1)<br>No (0)
28	Has a health professional ever told you that you have high cholesterol?	Yes (1)<br>No (0)
29	Has a health professional ever told you that you have kidney disease?	Yes (1)<br>No (0)

	Question	Response options (Frailty Index score weighting)
30	Has a health professional ever told you that you have a liver disease?	Yes (1)<br>No (0)
31	Has a health professional ever told you that you have macular degeneration in your eye?	Yes (1)<br>No (0)
32	Has a health professional ever told you that you have cataracts in your eyes?	Yes (1)<br>No (0)
33	Has a health professional ever told you that you have periodontitis – which means damage to the gums and bone around your teeth?	Yes (1)<br>No (0)
34	Has a health professional ever told you that you have dementia or Alzheimer's disease?	Yes (1)<br>No (0)
35	Has a health professional ever told you that you have depression?	Yes<br>No
36	Has a health professional ever told you that you have hearing loss?	Yes<br>No
37	Do you need to wear hearing aids?	Yes (-)<br>No (0)
38	Can you hear properly with your hearing aids in?*	Yes (0)<br>No (1)
39	Do you need glasses or contact lenses?	Yes (-)<br>No (0)
40	And can you see properly with glasses or contact lenses in?*	Yes (0)<br>No (1)
41	In the last month, how often did you have difficulties chewing any foods because of problems with your teeth, mouth, dentures or jaw?	Never (0)<br>Hardly (0)<br>Occasionally (0.5)<br>Fairly often (1)<br>Very often (1)
42	In the last month, how often have you had painful aching in your mouth?	Never (0)<br>Hardly (0)<br>Occasionally (0.5)<br>Fairly often (1)<br>Very often (1)
43	How is your appetite these days? Would you say it's normal, fair or poor?	Normal (0)<br>Fair (0.5)<br>Poor (1)
44	Is your appetite these days better, worse or about the same as usual?	Better<br>Worse<br>About the same
45	Have you been losing weight without meaning to?	Yes (1)<br>No (0)
46	How tall are you in centimeters? Please enter the number in the keypad. If you don't know, enter the number zero.	[Number entry]
47	How much do you weigh in kilograms? Please enter the number in the keypad. If you don't know, enter the number zero.	[Number entry]
48	Do you think you are underweight, normal weight, overweight, or obese?	Underweight<br>Normal Weight<br>Overweight<br>Obese
49	Over the last four weeks, how often have you been bothered by pain in your arms, legs or joints?	Not bothered (0)<br>Bothered a little (0.5)<br>Bothered a lot (1))

	Question	Response options (Frailty Index score weighting)
50	Over the last four weeks, how often have you been bothered by feeling tired of having low energy?	Not bothered (0)<br>Bothered a little (0.5)<br>Bothered a lot (1))
51	Over the last four weeks, how often have you been bothered by trouble sleeping?	Not bothered (0)<br>Bothered a little (0.5)<br>Bothered a lot (1))
52	Throughout our lives, most of us have had pain from time to time (such as minor headaches, sprains, and toothaches).<br>Today, have you had pain other than these everyday kinds?	Yes<br>No
53	Please show us the areas where you are feeling pain. You can do this by tapping the areas of your body where you feel pain. You can choose more than one area.*	[Tap relevant body part image]
54	Now, please show us the one area where you have the most pain, by tapping that area.*	[Tap relevant body part image]
55	Now, please rate your pain from zero to ten, by choosing the number that best describes your pain on AVERAGE in the past 24 hours. Zero is no pain. Ten is pain as bad as you can imagine*	[Number entry]
56	In the past year, how often did you have a drink containing alcohol?	Never<br>Monthly<br>2-4 times a month<br>2-3 times a week<br>More than that
57	Has a relative, friend, doctor or other healthcare worker been concerned about your drinking or suggested you cut down?	Yes – during the last year (1)<br>Yes – but not in the last year (0.5)<br>No
58	Do you need help from another person to feed yourself?*	Yes (-)<br>No (0)
59	How much help do you need?	I need a little help (0.5)<br>I can't do this without help (1)
60	Do you need help from another person to take a bath or shower?*	Yes (-)<br>No (0)
61	How much help do you need?	I need a little help (0.5)<br>I can't do this without help (1)
62	Do you need help from another person to dress yourself?	Yes (-)<br>No (0)
63	How much help do you need?	I need a little help (0.5)<br>I can't do this without help (1)
64	Do you need help from another person to go to the toilet?*	Yes (-)<br>No (0)
65	How much help do you need?	I need a little help (0.5)<br>I can't do this without help (1)
66	Do you need help from another person to get into or out of your bed?*	Yes (-)<br>No (0)
67	How much help do you need?	I need a little help (0.5)

	Question	Response options (Frailty Index score weighting)
		I can't do this without help (1)
68	I'd like you to answer as if you were in the community today. If you were in the community today, would you need help from another person to… use a phone to call someone? *	Yes (-)<br>No (0)
69	How much help would you need?	I need a little help (0.5)<br>I can't do this without help (1)
70	If you were in the community today, would you need help from another person to… Cook your own meals? *	Yes (-)<br>No (0)
71	How much help would you need?	I need a little help (0.5)<br>I can't do this without help (1)
72	If you were in the community today, would you need help from another person to.. Go shopping for your groceries?*	Yes (-)<br>No (0)
73	How much help would you need?	I need a little help (0.5)<br>I can't do this without help (1)
74	If you were in the community today, would you need help from another person to… Take the right medications you need, at the right time?*	Yes (-)<br>No (0)
75	How much help would you need?	I need a little help (0.5)<br>I can't do this without help (1)
76	If you were in the community today, would you need help from another person to… Travel from your home to a medical appointment?*	Yes (-)<br>No (0)
77	How much help would you need?	I need a little help (0.5)<br>I can't do this without help (1)
78	If you were in the community today, would you need help from another person to… Keep your home and belongings clean and tidy?*	Yes (-)<br>No (0)
79	How much help would you need?	I need a little help (0.5)<br>I can't do this without help (1)
80	Do you need something to help you walk, such as a walking stick or frame?	Walking stick =0.5<br>Frame =1<br>Wheelchair = 1<br>Don't need =0
81	Do you have any problems keeping your balance?	Yes (1)<br>No (0)
82	How many falls have you had in the past 3 months? If you haven't had any, press zero	[Number entry]<br><br>1 or more (1)<br>0 (0)
83	In the past 7 days, on how many days did you do vigorous activities?<br>These are activities that you're doing for more than 10 minutes, take a lot of hard physical effort and make you breathe much harder than normal. For example heavy lifting, running fast or digging.	[Number entry]

	Question	Response options (Frailty Index score weighting)
84	How long did you do these vigorous activities for, each day?	Less than 20 minutes<br>20-30 minutes<br>30 minutes – 1 hour<br>1-2 hours<br>2-3 hours<br>More than 3 hours
85	Do you have any problems with controlling your bladder or leaking?	Yes (1)<br>No (0)
86	Do you ever have any accidents with your bowels where you don't get to the toilet in time?	Yes (1)<br>No (0)
87	Do you have problems with your memory?	Yes (1)<br>No (0)
88	During the past year, how often did you read, like magazines, newspapers or books, including digital versions?	
89	During the past year, how often did you participate in programs or learning activities?	Every day or almost every day<br>Several times a week<br>Several times a month<br>Several times a year<br>Once a year or less
90	During the past year, how often did you play games, board games or puzzles, like cards or crosswords, including digital versions?	Every day or almost every day<br>Several times a week<br>Several times a month<br>Several times a year<br>Once a year or less
91	During the last year, how often did you do jobs, including unpaid ones, where you needed to use your thinking skills like planning how to do something or solving a problem or making something?	Every day or almost every day<br>Several times a week<br>Several times a month<br>Several times a year<br>Once a year or less
92	Over the last two weeks, how much have you felt relaxed?	None of the time (1)<br>Rarely (1)<br>Some of the time (0.5)<br>Often (0)<br>All of the time (0)
93	Over the last two weeks, how much have you felt like you've been thinking clearly?	None of the time (1)
94	Over the last two weeks, how much have you been feeling cheerful?	Rarely (1)
95	Over the last two weeks, how much have you felt optimistic about the future?	Some of the time (0.5)
96	Over the last two weeks, how much have you felt close to other people?	Often (0)

Note:*Question will skip or appear depending on response to relevant preceding question.
