# Supplementary material for: Equitable Digital Frailty Screening for Marginalized Older Adults Using Audio Computer-Assisted Self-Interview: Collaborative Development Guide and User Testing Study
Source: JMIR Form Res. 2026 May 13;10:e85768. doi: 10.2196/85768 (PMC13170664; doi:10.2196/85768)
Supplement: Multimedia Appendix 2 [file formative-v10-e85768-s002.docx]

**Multimedia Appendix 2: Development and User Testing Checklist for Equitable, ACASI-based Digital Health Screening Tools in Low-Resource Settings**

The following is a non-exhaustive list of suggested considerations for researchers, clinicians and developers embarking on similar projects entailing collaborative development of accessible, ACASI-based digital health screening tools for use in marginalized populations and resource-constrained environments.

**Key Principles Summary**

Equity-Centered: Address digital determinants of health at individual (literacy, trust), interpersonal (clinician relationships), community (infrastructure), and societal (design standards) levels.

Collaborative: Integrate expertise from researchers, clinicians, developers, end-users, and community representatives throughout all phases.

Context-Specific: Recognize that marginalized settings require unique adaptations; avoid one-size-fits-all approaches.

Iterative: Budget time and resources for multiple cycles of development, testing, and refinement.

Evidence-Based: Ground content and design through ongoing literature review.

Transparent: Document processes, governance, conflicts, and limitations clearly to build trust and enable replication.

**Part 1: Project Setup and Governance**

**Multi-stakeholder Governance**

☐ Assemble core research team with diverse expertise (clinical, epidemiological, technical, cultural)

☐ Identify and engage end-users early (clinicians, administrators, target population members)

☐ Define clear roles, decision-making authority, and consultation mechanisms for different stakeholders

☐ Establish advisory/reference groups for cultural oversight and community/end-user input

☐ Establish regular meeting cadences with varying engagement expectations across project phases

☐ Establish smaller "development subgroup" within core team for day-to-day decisions

☐ Communicate major updates to broader team through regular meetings and email

**Software Development Partnership**

☐ Select development partner (note: organisational proximity can enable frequent communication)

☐ Establish formal contract (consider covering scope, intellectual property, data management, security protocols, ongoing support)

☐ Include developer representative in core team meetings where appropriate

☐ Create shared documentation systems for streamlined communication (shared folders, naming conventions)

☐ Budget adequate time and resources for iterative development cycles

**Regulatory and Ethical Foundation**

☐ Obtain ethics approvals from all relevant jurisdictions and institutional bodies (applies where research is planned)

☐ Develop protocol and sign off from all team members (may include: project scope, governance and roles, methodological approach, ethical considerations, data management and storage, dissemination etc.)

**Part 2: Collaborative Design Process**

**Evidence-Informed Content Development**

☐ Conduct co-design sessions with target populations and stakeholders

☐ Conduct literature review of relevant topics (e.g., digital health, health equity, ACASI applications, target population needs)

☐ Ground project in relevant digital/health equity frameworks/HCI principles

☐ Hold structured consultation sessions with core team to establish "wish list" of domains and features

☐ Document rationale for inclusion/exclusion of specific items or features

☐ Seek ad hoc expert consultation on specific domains

**Iterative Refinement**

☐ Budget sufficient time for prototype development with multiple iteration cycles

☐ Develop beta versions for internal team testing and feedback

☐ Conduct informal testing with broader networks (family members, clinicians) before formal user testing

☐ Create systematic documentation for changes (spreadsheets tracking items, wording, scoring)

☐ Plan for formal user testing before validation study

**Part 3: Technical Design Features**

**Accessibility and Universal Design**

☐ Audio delivery: Provide clear audio narration for all questions and response options

☐ Simple visual design: Use consistent color scheme, sans-serif fonts, adequate contrast

☐ Icon support: Include clear, simple, universally recognizable icons for all content

☐ Device selection: Choose devices balancing screen size, weight, and readability

☐ Headphone provision: Provide noise-cancelling headphones for privacy and audio clarity

☐ Tutorial: Include interactive tutorial with practice opportunities; allow repetition as needed

☐ Test visual content for cross-cultural appropriateness and comprehension

**Interaction Design**

☐ Simple response mechanisms: Use tap-only interactions; avoid complex gestures

☐ Consistent layout: Fixed toolbar, progress bar, predictable screen structure

☐ Navigation control: Provide back, skip, and next buttons; allow audio replay

☐ Visual feedback: Highlight selected responses; provide audio confirmation for interactions

☐ Flexible pacing: Allow users to repeat questions/audio as often as needed before proceeding

**Content Adaptation**

☐ Use lay language, strengths-based wording, and avoid stigmatizing language

☐ Shorten questions to avoid screen crowding and improve comprehension

☐ Make conversational wording adaptations while maintaining validity

☐ Adapt context-specific items for institutional settings (e.g., IADLs "as if living in community")

☐ Balance comprehensiveness with completion time for users with varying literacy/cognitive capacity

☐ Select validated measures where possible; document any wording modifications

**Audio Development**

☐ Consider AI-generated voice where feasible

☐ Select voice informed by end-user needs (e.g., clarity, accent, pace, tone)

**Part 4: Data Management and Security**

**Infrastructure Considerations**

☐ Design for offline use with secure local data storage

☐ Weigh privacy and infrastructure benefits of offline storage against needs for real-time clinical access

☐ Anticipate context-specific security needs across deployment settings

**Clinical Deployment Planning**

☐ Assess whether data-at-rest encryption is required for clinical settings

☐ Plan for automated secure transfer mechanisms or health system integration

☐ Develop protocols with relevant IT teams (health services, correctional services)

☐ Implement audit trails for clinical deployment

☐ Recognise that security requirements vary by jurisdiction; plan for context-specific adaptation

**Part 5: User Testing and Evaluation**

**Recruitment and Sampling**

☐ Define inclusion criteria aligned with tool purpose (consider: age range, education levels, cultural backgrounds, cognitive abilities, relevant life experiences)

☐ Recruit through diverse pathways to achieve heterogeneous sample

**Testing Protocol**

☐ Allow adequate time and comfortable setting

☐ Observe users completing tool; note technical issues and usability problems

☐ Conduct structured feedback interviews covering: acceptability constructs, usability (e.g., SUS), specific design elements

**Analysis and Refinement**

☐ Analyze quantitative usability and acceptability data (descriptive statistics)

☐ Conduct content analysis of qualitative feedback for actionable improvements

☐ Test for missing data or completion issues

☐ Identify specific bugs or technical problems requiring developer attention

☐ Document user suggestions for future versions

☐ Plan iterative refinement before validation study in target setting

**Part 6: Transparency and Reporting**

**Conflicts of Interest**

☐ Disclose any team members with dual roles (e.g., co-investigator and developer)

☐ Clarify extent of developer involvement in authorship and interpretation

☐ Document intellectual property arrangements

**Limitations**

☐ Acknowledge limitations of community testing vs. target setting validation

☐ Distinguish between usability/acceptability testing and clinical validation

**Future Directions**

☐ Outline validation studies needed (comparison with clinician-administered tools, sensitivity/specificity)

☐ Describe implementation research requirements (workforce integration, resource needs)

☐ Identify longitudinal outcome studies to establish clinical impact

☐ Consider adaptation to other marginalized populations to test transferability
